# Massive hospital-wide bacillus outbreak related to hospital linen and construction

**DOI:** 10.1186/1753-6561-5-S6-O77

**Published:** 2011-06-29

**Authors:** M Balm, C Teo, R Jureen, R Lin, D Fisher

**Affiliations:** 1 National University Hospital, Singapore, Singapore

## Introduction / objectives

At the National University Hospital in Singapore, the baseline average number of bacillus cultures/month is eight. An outbreak became evident when 274 clinical isolates of *Bacillus* were recovered from 230 inpatient episodes between April and August 2010. An investigation was undertaken.

## Methods

Chart reviews of affected patients and extensive environmental sampling was followed by a review of hospital ventilation systems, cleaning protocols and laundry processes. Response to interventions was monitored via clinical case numbers and environmental sampling over a six month period.

## Results

*B. cereus* complex constituted 164 cases (71.3%). Bacteraemia comprised 207 patient episodes (90.0%), of which 124 occurred in immunocompromised patients or those with intravascular devices. Physicians treated the organism in 68 episodes (29.5%). Environmental investigations confirmed heavy air contamination particularly within patient rooms and air conditioned wards. Dense airborne contamination outside the hospital adjacent to large earthworks on a construction site was demonstrated (~600CFU/m^3^). Towels were heavily contaminated even after laundering (7403±1054 spores/cm^2^). Amplification of spores occurred in clean linen due to storage conditions (165±84 spores/cm^2^ pre-storage vs 4437±1228 spores/cm^2^ post-storage). Interventions focusing on laundry protocols, environmental cleaning and air filtration saw clinical case numbers return to baseline levels within three months.

## Conclusion

Environmental contamination with *Bacillus* may be an under-recognised infection risk in hospitals exposed to construction work. Laundering and environmental cleaning processes that are not sporicidal carry a greater risk. Storage conditions of cleaned linen can amplify *Bacillus* contamination.

## Disclosure of interest

None declared.

